# Clinical experience of use of percutaneous peripheral arterial cannulation in sick neonates in a developing country

**DOI:** 10.1186/s12887-021-02943-2

**Published:** 2021-11-02

**Authors:** Sachin Shah, Amita Kaul, Shambhavi Mishra, Shridhar Pawale

**Affiliations:** 1 Department of Neonatal and Pediatric Intensive Care Services, Surya Mother and Child superspecialty Hospital, Octroi Naka,Sr.No.8, Bhujbal Chowk, Near Wakad, Pune, Maharashtra India; 2 Department of Neonatology, Surya Mother and Child Superspecialty Hospital, Pune, Maharashtra India

**Keywords:** Arterial cannulation, Neonates, Vascular catheterization

## Abstract

**Background:**

Continuous invasive arterial monitoring is necessary in sick neonates needing hemodynamic and ventilatory support. The primary objective of our study was to describe clinical experience with percutaneous peripheral arterial cannulation (PAC) in sick neonates.

**Methods:**

Neonates needing PAC were prospectively enrolled in the study. Inclusion criteria were: neonates needing respiratory support (invasive or non-invasive), neonates requiring vasoactive medications or neonate likely to need more than 5 sampling pricks in 24 h.

**Results:**

One hundred eight neonates (93.1%) needed cannulation of one arterial site while 8 (6.9%) needed cannulation of 2 arterial sites, thus giving a total of 124 cannulations. Out of the 124 cannulations, 102 (82%) were performed in first attempt, while 22 (18%) cannulations needed 2 or more attempts. Serious complications like discolouration of digits, blanching of skin or bleeding were seen in 6 (4.9%) cannulations. These resolved after removal of arterial line and no long term consequences were noted. On comparing neonates having radial arterial cannulation(*n* = 108) with posterior tibial arterial cannulation (*n* = 16) there was no difference in duration of arterial line between radial artery group (mean, SD 53.30 ± 22.56) and posterior tibial artery group (mean, SD 48.25 ± 27.39). However, more attempts were needed to cannulate post tibial artery (mean, SD 2.25 ± 1.32) as compared to radial artery (mean 1.22 ± 0.789) and this difference was statistically significant (MD -1.02, 95% CI − 1.75 to − 0.30). There was no difference in incidence of serious complications between the radial artery group (3.7%, *n* = 4) as compared to posterior tibial group (5.5%, *n* = 1, OR 0.57, 95% CI 0.06–5.51, *p* = 0.63).

**Conclusions:**

Peripheral arterial cannulation is a safe method for hemodynamic monitoring and blood sampling in sick neonates. Complications can be minimized by diligent monitoring and proactive removal of line if there is damping of tracing.

**What is known**: Umbilical arterial line is an routine method of invasive monitoring in NICU but it is associated with high chances of thrombosis.

**What is new**: Peripheral arterial line, is fairly simple,cost effective procedure for invasive monitoring of high risk neonates with minimal side effects.

## Background

Sick neonates admitted in the Neonatal Intensive Care Unit (NICU) need frequent monitoring in form of arterial blood gases, laboratory studies and continuous blood pressure monitoring. Intermittent sampling by arterial puncture, venipuncture or capillary puncture can lead to excessive handling of neonate, pain, local hematoma, thrombosis, etc. contributing to significant morbidity [1]. Also, non-invasive blood pressure (NIBP) monitoring does not correlate with invasive blood pressure making judgements based on NIBP erroneous [2]. Hence, inspite of advances in non-invasive monitoring, continuous arterial monitoring is still necessary in sick neonates, especially those needing hemodynamic and ventilatory support. The umbilical artery is the most commonly used vessel, but percutaneous peripheral arterial cannulation (PAC) is a safe alternative method [3].Despite extensive description of percutaneous peripheral arterial cannulation in children and adults, there are very few reports in neonates [4, 5].

The primary objective of this study was to measure the risk of serious complications after percutaneous arterial cannulation in sick neonates. The secondary objective was to measure the difference in rate of complications between two different sites of cannulation and among different birth weight groups.

## Methods

The study was carried out in a tertiary care 35 bed NICU of urban India from 1st August 2019 to 30th September 2020 and was approved by the institutional ethics committee. Informed written consent was taken from the parents. Neonates needing percutaneous peripheral arterial cannulation were prospectively enrolled in the study.

### Inclusion criteria

All neonates needing percutaneous peripheral arterial cannulation.

### Exclusion criteria

Neonates with birth weight < 750 g.

Indications for PAC were as follows:

1. Neonates needing respiratory support (invasive or non-invasive)
2. Neonates requiring vasoactive medications
3. Neonate likely to need more than 5 sampling pricks in 24 h

Primary outcome measure was the incidence of complications after percutaneous peripheral arterial cannulation.

Secondary outcome measures were duration of PAC line and number of attempts for PAC.

### Procedure

The clinicians were free to choose between umbilical arterial cannulation or peripheral arterial cannulation. If PAC was the chosen mode then, percutaneous radial artery cannulation was always attempted first. Before puncture, skin was cleaned with 2.5% chlorhexidine gluconate plus 70% ethanol swab and left to dry. Allen’s test was not done prior to cannulation. The puncturing technique was left to discretion of the clinician and both single wall and transfixation techniques were used, Transillumination use was also at the discretion of clinician. A 24 G or 26 G cannula (BD Neoclon Pro IV cannula OR Vycan, Vygon) was used. After successful cannulation, it was then connected to an extension tubing which was in turn connected to transducer (Edward Lifesciences services GmbH, Germany) and multipara monitor (Draeger Vista 120, Draeger, Germany) for continuous blood pressure monitoring. The arterial line was kept patent by infusing 0.45% NS or 0.9% NS diluted 1:1 with heparin at the rate of 0.6–1 ml/hour.

The catheter was taped using a transparent dressing in such a way that the puncture and catheter site was visible and also the fingers/digits were left exposed for inspection. Maximum of two attempts at one site and 2 sites were permitted. Failure to cannulate 2 sites was classified as procedural failure. Repeat cannulation of already punctured arterial sites was not allowed but different site could be attempted after a rest period of 12–24 h. All the cannulations were done either by the neonatology fellow or inhouse NICU consultant. Decision to abort PAC attempt and insert umbilical lines could be taken by the attending neonatologist depending on the clinical condition of the neonate.

The arterial line was used only to obtain blood for laboratory studies and blood gas analysis. No medications, additional fluids, electrolytes, or blood products were given through the line. Use of the catheter was discontinued under the following conditions:

1. persistent skin blanching or erythema over the catheter tip,
2. discoloration of the fingers,
3. Inability to withdraw blood or loss of the blood pressure tracing
4. leakage around the insertion site.

After catheter removal, firm pressure was applied to the insertion site with a sterile gauze pad for approximately 5 min to ensure hemostasis.

The demographic characteristics, indications for PAC, number of arterial attempts, duration of peripheral arterial cannulation and complications were prospectively recorded. Discolouration of digits, blanching of skin or bleeding were classified as serious complications. Subgroup analysis was performed to compare radial arterial cannulation group and posterior tibial arterial cannulation group and to study effect of PAC across various birth weight groups.

### Sample size calculation

For calculation of sample size, we assumed a baseline risk of complications (based on literature search) as 10% in neonates needing peripheral arterial cannulation. We hypothesized that to detect such a difference with 5% margin of error and 95% confidence, we would need a minimum sample of 111 neonates.

### Statistical evaluation

We used SPSS version 23 (IBM Inc., USA) for statistical analysis. Continuous variables are presented either as mean ± standard deviation (SD). We tested differences between groups with analysis of variance (ANOVA) or Student’s t test. Categorical variables are presented as counts (percentages). We used the chi-square test to compare frequency distributions between two categorical variables. A *P* value of less than 0.05 was considered significant.

## Results

There were 541 admissions to NICU, out of which 141 were eligible for arterial line insertion. Umbilical arterial line was inserted primarily in 21 neonates while 4 neonates had PAC failure and hence umbilical catheterization was performed. Peripheral arterial cannulation was performed successfully in 116 neonates.

One hundred eight neonates (93.1%) needed cannulation of one arterial site while 8 (6.9%) needed cannulation of 2 arterial sites since the first one had to be removed prematurely and there was ongoing need for arterial line, thus giving a total of 124 cannulations (Table 1).

**Table 1:** Demographic characteristics of neonates included in the study

Total number of neonates studied (n)	116
Total number of peripheral arterial cannulations (PAC)	124
Gestational age (Mean, SD)	32.8 ± 3.5
Birth weight (Mean, SD)	1870.48 ± 741.41
PAC as per gestational age	
GA < 28 weeks	16 (12.9%)
GA 28–31 + 6 weeks	40 (32.2%)
GA 32–36 weeks	52 (41.9%)
GA > 36 weeks	16 (12.9%)
PAC as per birth weight	
BW 750–1000 g	8 (6.4%)
BW 1001–1500 g	44 (35.4%)
BW 1501–2500 g	52 (41.9%)
BW > 2500 g	20 (16.1%)
Total number of arterial lines inserted	
Radial arterial line	108 (87.1%)
Ulnar arterial line	0
Posterior tibial arterial line	16 (12.9%)
Duration of peripheral arterial line, (mean, SD)	52.65 ± 23.17
Number of infants on vasoactive medications	26 (22.4%)
**Indication for PAC**	
BP monitoring and titration of vasoactive medications	26 (20.9%)
Sampling	98 (79.1%)
**Reason for removal of PAC**	
Elective removal due to lack of need for PAC	98 (79%)
Does not bleed back Or Dampening of tracing	20 (16.1%)
Non elective removal due to complications	6 (4.9%)

Out of the 124 cannulations, 102 (82%) were performed in first attempt, while 10 (8.1%) were performed on 2nd attempt. Eight cannulations (6.5%) needed 3 attempts, while 4 (3.2%) cannulations were performed on 4th attempt.

24G cannula was used in 117 (94.3%) cannulations while 26G cannula was used in remaining 7 (5.7%) cannulations. Cold light source for visualization of artery was used in 90 (72.4%) cannulations.

The demographic and clinical characteristics are presented in Tables 1 and 2.

**Table 2:** Clinical conditions needing PAC insertion (*n* = 116)

Respiratory Distress Syndrome (RDS)	94 (81%)
Asphyxia	4 (3.4%)
Meconium aspiration syndrome (MAS)	10 (8.6%)
Persistent pulmonary hypertension of newborn (PPHN)	14 (12%)
Sepsis	22 (19%)
Surgical (Congenital diaphragmatic hernia, volvulus, Necrotising enterocolitis), etc)	6 (5.1%)
Other	12 (10.3%)

Serious complications like discolouration of digits, blanching of skin or bleeding were seen in 6 (4.9%) neonates. (Table 3). Discolouration and blanching resolved after removal of arterial line and warming of contralateral limb, while the bleeding stopped after application of local pressure. No long term consequences were noted. Rate of complications was higher in posterior tibial arterial cannulation as compared with radial arterial cannulation (Table 4).

**Table 3:** Complications associated with Peripheral arterial cannulation (Total cannulations = 124)

Complications, n (%)	26 (20.9%)
Blanching of skin	3 (2.4%)
Dampening of tracing leading to removal of line	14 (11.3%)
Inability to bleed back	6 (4.9%)
Local Swelling	0
Leakage or bleeding around insertion site	1 (0.8%)
Accidental dislodgement	0
Discoloration of digits	2 (1.6%)
Local infection	0

**Table 4:** Comparison of complications amongst Radial versus posterior tibial arterial cannulation

	Radial arterial line (n = 108)	Posterior tibial art line (n = 16)	P value	Mean difference (MD), 95% Confidence intervals (95% CI)
GA (mean, SD)	32.67 ± 3.67	34.13 ± 2.33	0.042	−1.48 (−2.85 to −0.590
BW (mean, SD)	1817.50 ± 742.94	2228.13 ± 643.10	0.029	410.62 (− 776.12 to −45.125)
Number of attempts	1.22 ± .789	2.25 ± 1.32	0.008	− 1.02 (− 1.75 to − 0.30)
Duration of PAC (mean, SD)	53.30 ± 22.56	48.25 ± 27.39	0.491	5.04 (− 10.04 to 20.13)
Complications *Inability to bleed back* *Dampening of tracing* *Blanching* *Discolouration of digits* *Leakage around insertion site*	18 (14.5%) 3 10 3 1 1	8 (50%) 3 4 0 1 0	0.001	

ANOVA test showed that there was significant difference in the number of attempts to insert arterial cannula between the 4 weight categories (*p* = 0.035) with the smallest weight category needing more attempts. The duration of arterial cannulation was also significantly different amongst the groups (*p* = 0.016), the duration being higher in neonates less than 1000 g and those more than 2500 g. (Table 5).

**Table 5:** Comparison of complications of peripheral arterial cannulation across various BW categories

	BW 750–1000 g *N* = 8	BW 1001–1500 g *N* = 44	BW 1501–2500 g *N* = 52	BW > 2500 g *N* = 20
Number of attempts Mean, SD	2.25 ± 2.31	1.2 ± 0.59	1.33 ± 0.87	1.4 ± 0.68
Duration of PAC Mean, SD	73.25 ± 13.55	50.77 ± 24.53	48.42 ± 22.47	59.5 ± 20.16
Complications	2	11	10	3
*Inability to bleed back*	0	4	2	0
*Dampening of tracing* *Blanching*	2 0	5 1	6 1	1 1
*Discolouration of digits*	0	1	0	1
*Leakage around insertion site*	0	0	1	0

## Discussion

In our prospective study we showed that percutaneous peripheral arterial cannulation is a safe method for sampling purposes and for monitoring blood pressure in sick neonates. Majority of the eligible neonates were successfully cannulated in first attempt. Also, over 90% of the neonates needed cannulation of a single site. Incidences of major complications were low.

Arterial lines were electively removed in 79.1% neonates while in remaining 20.9%, the lines had to be removed due to complications. The commonest complications seen with PAC were damping of trace leading to removal of line or inability of the line to bleed back. No adverse effects were noted after removal of such lines. Serious complications like discolouration of digits, blanching of skin or bleeding were seen in 6 (4.9%) neonates. Discolouration and blanching resolved after removal of arterial line and warming of contralateral limb, while the bleeding stopped after application of local pressure. No long term consequences were noted till discharge from NICU.

On comparing neonates having radial arterial cannulation with posterior tibial arterial cannulation there was no difference in the birth weight, gestational age or duration of arterial cannulation between the 2 groups. However, more attempts were needed to cannulate post tibial artery as compared to radial artery and this difference was statistically significant. This could be due to the fact that clinicians are less familiar with technique of posterior tibial artery cannulation and it is also not the first choice for cannulation. Though the incidence of complications was high in posterior tibial arterial cannulation, majority were minor (1 neonate had discolouration of digits while remaining 7 had the line removed due to damping of trace or inability to bleed back). The number of posterior tibial cannulation was small and hence these findings should be interpreted with caution.

On analyzing the cannulations according to birth weight, it was noted that most of PACs were performed for Low birth weight (LBW) and Very low birth weight (VLBW) neonates. Extremely low birth weight neonates (ELBW) formed a small percentage of this population since they preferably received umbilical arterial cannulation. Interestingly the duration of arterial line was higher in ELBW neonates and early term/term neonates. This could be due to the fact that VLBW/LBW neonates were relatively less sick and had earlier elective removal of arterial line once the primary condition like RDS resolved. Line damping and inability to bleed back was more common in the ELBW group but there was no difference in the incidence of serious complications. Also more number of attempts were needed to cannulate ELBW neonates, but were still within acceptable limits. We could not find a reference for “number of acceptable attempts” but most of units would consider 2–3 attempts as permissible.

The relatively high success rate of cannulation and lower rate of complications could be due to competence in the technique and unit familiarity.

In our experience the earliest sign of problem with PAC is damping of tracing on the monitor. This could be due to arterial vasospasm. If this vasospasm does not resolve after few saline flushes or warming of contralateral limb, it is prudent to remove the line. Persistence with a line with dampened tracing may lead to vascular complications. In our unit we are proactive with line removal if we feel that damping is due to vasospasm.

There are very few recent reports of peripheral arterial cannulation. In a study published in 1987 Randel et al. [6] analysed 158 peripheral arterial cannulations and the complications that they noted were absence on blood return in 38 (24%) neonates, blanching in 12 (7.6%), leaking in 9 (5.7%), accidental dislodgement in 3 (1.9%) and swelling in 3 (1.9%) neonates. We could not find published larger studies analyzing the use of PAC in a developing country.

In most of the NICUs across the world, umbilical arterial catheters (UAC) are preferred. But, UACs are not without their own set of complications [7]. In a recent systematic review, Rizzi et al. [8] found that the incidence of UA related thrombosis is about 20% in neonates and the symptoms included limb ischemia, Hypertension, hematuria, NEC, etc.

It is possible that peripheral arterial cannulation is perceived to be technically more difficult or the fear of vascular complications may preclude its use. But in skilled hands and with use of transillumination, the success rate could be high as it is operator dependent.

The cost of PAC (intracath, angiocath) is also quite economical as compared to umbilical artery catheter. The cost of insertion of PAC works around to Rs 4134 (~ 55 USD) while that of insertion of umbilical arterial catheter works out to Rs 6750 (~ 90 USD). Also, umbilical venous cannulation is done simultaneously with UAC which will increase the cost by additional 30%.

## Conclusions

Peripheral arterial cannulation is a safe method for hemodynamic monitoring and blood sampling in sick neonates. Complications can be minimized by diligent monitoring and proactive removal of line if there is damping of tracing.

## Data Availability

The datasets used and or analysed during the study are available from the corresponding author on reasonable request.

## Abbreviations

PAC: Peripheral arterial line
NIBP: Non invasive blood pressure
VLBW: Very low birth weight
LBW: Low birth weight
ELBW: Extremely low birth weight
